# Electrospun PVA/Chitosan Nanofibers Containing AgNPs and β-Sitosterol Exert Superior Antibacterial Effect

**DOI:** 10.3390/nano16211438

**Published:** 2026-11-06

**Authors:** Talal A. Almohaya, Salman Alrokayan, Tajamul Hussain

**Affiliations:** 1 King Abdulaziz City for Science and Technology, Riyadh 11451, Saudi Arabia; 444105659@student.ksu.edu.sa; 2 Biochemistry Department, College of Science, King Saud University, Riyadh 11451, Saudi Arabia; salrokayan@ksu.edu.sa; 3 Center of Excellence in Biotechnology Research, King Saud University, Riyadh 11451, Saudi Arabia

**Keywords:** electrospinning, poly vinyl alcohol, chitosan, silver nanoparticles, beta-sitosterol, nanofiber, antibacterial, cytotoxicity

## Abstract

Due to their well-documented robust antibacterial properties, silver nanoparticles (AgNPs) are commonly used in wound dressings to prevent bacterial infection. β-sitosterol (BST), a plant-derived phytosterol, further supports wound healing processes through its antioxidant, anti-inflammatory, and proangiogenic properties. In this study, we constructed a novel delivery system for its potential use in wound healing. Polyvinyl alcohol/chitosan (PVA/CS) nanofibers were prepared incorporating both BST and AgNPs by electrospinning method. This synthesized PVA/CS/AgNPs/BST nanofiber system was found to comply with the desired size, morphology, encapsulation efficiency, and time course of release for incorporated components, as confirmed by SEM, TEM, FTIR, HPLC, and XRD analyses. Zone of inhibition (ZOI) test revealed a significantly greater bacterial growth inhibition for constructed nanofiber systems than for AgNPs alone or PVA/chitosan blank fibers. Individually, AgNPs and AgNPs + BST demonstrated significant antibacterial activities against *Staphylococcus aureus* strains ATCC 29213, 43300, and BAA-977, with minimum inhibitory concentration (MIC) values of 62.5, 62.5, and 31.2 µg/mL, respectively. Likewise, AgNPs and AgNPs + BST showed antibacterial activities with MICs of 31.2 µg/mL and 15.6 µg/mL against *Pseudomonas aeruginosa* strains ATCC 27853 and MDR-7067 respectively. In contrast, no bacterial growth inhibitory effects were noted for BST against any of the studied bacterial strains. Compatibility analysis revealed that HFF1 cells treated with AgNPs and AgNPs + BST showed no cytotoxic effect at least up to concentrations of 15.6 µg/mL and 31.2 µg/mL, respectively, whereas BST alone had no effect on cell viability, even at higher concentrations. Collectively, these data show that the PVA/chitosan/AgNPs/BST system exercised a greater bacterial growth inhibitory effect against both Gram-positive and Gram-negative bacteria, underscoring its potential application in wound healing, subject to its in vivo validation.

## 1. Introduction

Electrospinning technology has been broadly implemented in the biomedical field in the construction of nanofiber-based drug delivery systems for wound healing and tissue regeneration. These electrospun nanofibers have become popular due to their ability to reproduce the structures that mimic natural extracellular matrices (ECMs) [1]. In addition, wound healing processes benefit from the properties of electrospun nanofibers, such as high surface-to-volume ratio, porosity, and flexibility [2]. These fibers also promote essential cell attachment, growth, and differentiation, absorption of exudate from wounds, and sustain moisture conditions suitable for healing [3]. They establish microbial resistance barriers and promote natural body healing processes simultaneously.

Nanofibers can be synthesized using natural and/or synthetic polymers [4]. Natural polymers including collagen, gelatin, chitosan, fibrinogen, fibronectin, hyaluronic acid, cellulose, and alginate are usually biocompatible and biodegradable and have been shown to facilitate wound healing [5,6]. The biodegradable synthetic polymers that are currently in use include PVA, PCL, PLA, PLGA, PU, and PEO/PEG [7]. While currently used nanofibers are synthesized from natural or synthetic polymers, these dressings often suffer from poor mechanical properties [8]. This limitation can be negated through crosslinking with synthetic polymers [8]. Different forms of wound dressing materials, including films, sponges, hydrogels, bandages, exosomes, foams, wafers, nanofibers, and nanofibrous materials such as nanofibrous mats, patches, films, and membranes, can be prepared using polymers [9]. However, the efficacies of these wound dressing nanofibers are marred by their poor antimicrobial properties [10]. As nanoparticles display an excellent antibacterial activity, they can be incorporated into wound dressings to prevent bacterial infection and improve the wound healing process [11]. Several nanoparticle types described to be suitable for wound dressings including metal-based nanoparticles, polymeric nanoparticles, and lipid-based nanoparticles [11]. Electrospun nanofibrous mats containing antibacterial nanomaterials are currently attracting attention in wound healing research, as they allow for easy gas exchange at the wound site, display a sustained and controlled release of loaded bioactive molecules and prevent bacterial infections, thereby accelerating wound healing [12,13,14].

Among the antibacterial nanoparticles, AgNPs are an ideal choice for wound healing as they are inexpensive, chemically stable, conduct electricity well, act as catalysts, and are resistant to a wide range of pathogens [15,16,17]. Silver has been recognized as a strong antibacterial metal that can effectively target multidrug-resistant pathogens and biofilm-forming bacteria, which are often found in chronic wounds. The size, shape, dose, and stabilizing agents used in the synthesis all play a role in how well an antibacterial agent works [18,19,20]. Importantly, several studies have demonstrated that AgNPs perform better as an antibacterial agent following their incorporation into nanofibers or other formulations.

BST, a plant-derived phytosterol with anti-inflammatory, antioxidant, and proangiogenic properties [21,22], has received increasing scientific attention due to its use as a healing and scar-reduction agent in topical treatments [23]. Topical BST application enhanced burn wound healing by increasing collagen synthesis and aiding tissue regeneration [24]. Importantly, several studies have shown the BST to exercise antibacterial effects either used alone or in combination with other bioactive agents [25,26,27,28]. Thus, utilizing the pro-wound-healing properties of BST by incorporating it alongside AgNPs into nanofiber-based wound dressings could be a potential strategy for accelerating wound healing. Towards this end, we constructed a novel delivery system by incorporating BST and AgNPs into PVA/chitosan nanomats by electrospinning method. Importantly, these bioactive agents have proven to perform better following their incorporation into nanofibers or other formulations due to their sustained and controlled release.

## 2. Materials and Methods

### 2.1. Materials

Low-molecular-weight CS, PVA, BST, FBS, penicillin, streptomycin, DMSO, and acetic acid (99.8%) were obtained from Sigma-Aldrich (St. Louis, MO, USA). AgNPs, (15 nm, 5000 ppm, Mono Nanopowder, Tawny) were obtained from US Research Nanomaterials (US Research Nanomaterials Inc., Houston, TX, USA), while the CellTiter 96^®^Aqueous One Solution Cell Proliferation Assay kit was procured from Promega (Southampton, UK). HFF-1 (SCRC-1041™; human foreskin fibroblast) cells, *Pseudomonas aeruginosa* strain ATCC 27853 and *Staphylococcus aureus* strains (ATCC 29213, ATCC 43300 and ATCC BAA-977) were obtained from ATCC (Manassas, VA, USA). *P. aeruginosa* strain, MDR 7067 was sourced from clinical repository. Mueller–Hinton agar and Mueller–Hinton broth were purchased from Scharlau (Barcelona, Spain), while methanol and ethanol (absolute, 99.8%) were obtained from Fisher Scientific UK Ltd. (Loughborough, UK). Acetonitrile (ACTN, ≥99.9% for HPLC) was purchased from Scharlau (Barcelona, Spain). Dulbecco’s Modified Eagle Medium (DMEM) was purchased from Capricorn Scientific (Ebsdorfergrund, Germany).

### 2.2. PVA and Chitosan Preparation

The CS was prepared (2% *w*/*v*) by dissolving CS powder in a 2% (*v*/*v*) aqueous acetic acid solution by magnetic stirring at 500 rpm overnight. PVA solution (10% *w*/*v*) was prepared by dissolving PVA powder in distilled water by stirring at 80–90 °C for 3 h to ensure its complete dissolution. CS and PVA solutions were mixed at 80/20, 70/30, 60/40, and 50/50 proportions (*v*/*v*) and stirred at room temperature to achieve homogeneity. The employed needle had a diameter of 1.2 mm; the needle tip-to-collector distance was maintained at 15 cm; the flow rate was adjusted to 0.5 mL/hour; and the applied voltage was set between 12, 16, 17, and 17 kV corresponding to 80/20, 70/30, 60/40, and 50/50 ratios of PVA/CS, to acquire a stable jet. The different electrospinning voltages used for the investigated PVA/chitosan ratios depended on the viscosity of the solvent polymer. For example, PVA/CS 50/50 has a higher viscosity than 80/20; the voltage ranges for reaching the Taylor cone are different, which critically affects the stability of the jet during electrospinning. The electrospun mats were assigned as PVA/CS 80/20, PVA/CS 70/30, PVA/CS 60/40, and PVA/CS 50/50. The details of PVA and CS volumes, concentrations and ratios used in the preparation of nanofibers are presented in Table 1.

### 2.3. Loading of BST and AgNPs into PVA/CS Fibers

BST and AgNP-loaded PVA/CS nanofibers were prepared using a Spraybase^®^ electrospinning instrument (Spraybase^®^, Dublin, Ireland). BST, at a concentration of 11 mg/mL, was dissolved in ethanol by gentle heating and magnetic stirring at room temperature for 3 h. AgNPs were kept at a 15 nm dispersion in water (5000 ppm). The PVA/CS (80/20) solution prepared as described above was stirred at room temperature for at least 1 h. BST and/or AgNPs, each at 0.43 wt.% relative to the total polymer mass (PVA/CS), were separately added to the PVA/CS polymer solution and electrospun to obtain PVA/CS/BST, PVA/CS/AgNPs and PVA/CS/BST/AgNPs nanofibers. The needle used in electrospinning had a diameter of 1.2 mm; the needle tip-to-collector distance was maintained at 20 cm; the flow rate was set to 0.5 mL/hour; and the applied voltage was 17 kV to acquire a stable jet.

### 2.4. Determinization of Morphological Characterization of Fibers

#### 2.4.1. Scanning Electron Microscopy (SEM)

Fiber morphology was examined using SEM (JSM-IT500HR SEM, JEOL Inc., Peabody, MA, USA). Fiber samples collected on aluminum foil, a conductive material, were placed on a metal stub without coating for SEM at an accelerating voltage of 5 kV. The fiber diameter was measured using the microscope’s software (SEM Operation: 3.010, version 1.010, JEOL TECHNICS Ltd., Tokyo, Japan), and the ImageJ program (version 1.54p) was used to measure the average diameter of at least 30 fibers to assess the fiber batch.

#### 2.4.2. Transmission Electron Microscope (TEM)

A TEM was used to assess the morphology, localization, and distribution of AgNPs within and on the fiber. A thin layer of electrospun fibers was collected directly onto TEM grids during electrospinning and then examined using a JEM-1011 transmission electron microscope (Pleasanton, CA, USA) at 100 kV; the magnification ranged from 50X to 600,000X (TEM-JEOL (JEM-1011)).

#### 2.4.3. Characterization by X-Ray Diffraction (XRD)

XRD was used to determine the structural changes that occur during drug loading into the fiber, as well as the crystalline or amorphous nature of the drug. XRD analysis was performed on PVA, CS, BST, and AgNPs, the corresponding physical mixture, blank PVA/CS fibers, and AgNPs/BST-loaded fibers. The physical mixture was prepared using the same relative mass proportions of the components present in the final drug-loaded fiber formulation. Prior to XRD analysis, AgNPs were dried for 12 h at 90 °C. XRD patterns were obtained using a Rigaku MiniFlex 600 (Tokyo, Japan), with Cu Kα radiation (λ = 1.5406 Å). The samples were mounted on a glass sample holder and analyzed at 40 kV, 15 mA, and a scan speed of 10°/min. The patterns were recorded in a 2θ range of 0 to 80°.

#### 2.4.4. Fourier-Transform Infrared Spectroscopy (FTIR) Analysis

A Thermo Smart ATR IS20 Spectrometer (Thermo Fisher Scientific, Waltham, MA, USA) FTIR analyzer was used to identify the functional groups, chemical interactions, and possible molecular interactions of drug-loaded and blank fibers, as well as of pure PVA, CS, BST, AgNPs, and their physical mixture (PVA/CS and PVA/CS/BST/AgNPs). The components were mixed according to the same relative mass proportions used in the final drug-loaded formulation. A spectral resolution of 4 cm^−1^ was used to obtain FTIR spectra of the samples in the wavenumber range 600–4000 cm^−1^, and 32 scans were taken for each sample. Spectra were plotted and analyzed using OriginPro 2025.

#### 2.4.5. UV–Visible Spectrophotometric Analysis

UV–Vis analysis was used to measure the absorption of AgNPs and quantify their amount. A calibration curve was prepared using AgNP standard dispersions in 40% ethanol at concentrations of 25, 12.5, 6.25, 3.125, 1.56, and 0.7 µg/mL. Three independently prepared fiber batches were analyzed, with three samples obtained from each batch (n = 9). The weight of each fiber sample was 5 mg PVA/CS/AgNPs/BST dispersed in 10 mL of 40% ethanol for 4 h to extract the AgNPs. The solution was measured using a LAMBDA 850+ UV/Vis spectrophotometer (PerkinElmer, Waltham, MA, USA) over the wavelength range of 200 to 800 nm to determine the characteristic maximum absorption wavelength (λmax) of the AgNPs. A 40% ethanol solution was used as a spectrophotometric blank.

#### 2.4.6. Analytical Quantification Using High-Performance Liquid Chromatography (HPLC)

High-performance liquid chromatography (HPLC) was used to detect and quantify BST using the Shimadzu LC-2030c 3D, PDA: LC-2030/2040 instrument (Kyoto, Japan). Chromatographic separation was performed using a Poroshell 120 EC-C18 column (150 × 3.0 mm, 2.7 micron) with a wavelength of 210 nm (Agilent Technologies, CA, USA). The mobile phase consisted of methanol (MeOH) and acetonitrile (ACN) 70:30 (*v*/*v*). The injection volume was 10 μL; the flow rate for the chromatographic separation was 1.5 mL/min with a 12 min runtime; and the column temperature was 20 °C. Standard solutions of beta-sitosterol were prepared in ethanol and serially diluted to 200, 100, 50, 25, 12.5, 6.25, and 3.125 µg/mL for calibration.

### 2.5. Determination of Drug Loading (DL) and Encapsulation Efficiency Percentage (EE%) 

The DL and EE% of BST-loaded fiber were assessed by dissolving the fiber weight (4 mg, n = 3) in 8 mL of 40% ethanol for four hours at ambient temperature to ensure complete nanofiber dissolution. The amount of BST was measured using the previous HPLC method. The AgNPs were assessed by dissolving the fiber weight (3 mg, n = 3) in 3 mL of 40% ethanol for four hours at ambient temperature to ensure complete nanofiber dissolution. The amount of AgNPs was measured using the UV/vis spectrophotometer method. The *DL* and *EE*% were calculated according to the following equations:

$$\mathrm{DL}=\frac{\mathrm{entrapped}\;\mathrm{drug}\;\mathrm{amount}}{\mathrm{weight}\;\mathrm{of}\;\mathrm{the}\;\mathrm{fibers}}$$

$$\mathrm{EE}\%=\frac{\mathrm{actural}\;\mathrm{drug}\;\mathrm{amount}}{\mathrm{theoretical}\;\mathrm{drug}\;\mathrm{amount}}\times 100$$

### 2.6. In Vitro Drug Release Assessment

The release of AgNPs and BST from the nanofibers was performed by weighing β-sitosterol/AgNP-loaded nanofibers (20 mg) and immersing them in glass vials containing 10 mL of pre-warmed 40% ethanol (pH 7) at 37 °C. The samples were incubated in a thermostatic shaker incubator set at 37 °C and 100 rpm and collected 1 mL samples at 1, 5, 10, 15, 30, 60, 120, 240, 360 min time points. The removed 1 mL of sample was immediately replaced with an equal volume of pre-warmed 40% ethanol to maintain a constant release-medium volume. The amount of released β-sitosterol and AgNPs was quantified using HPLC and UV–Vis spectrophotometry respectively. The cumulative percentage release of β-sitosterol and AgNPs was calculated as a function of time using the following equation:

$$\mathrm{cumulative}\;\mathrm{release}\;\%=\frac{\mathrm{cumulative}\;\mathrm{drug}\;\mathrm{amount}}{\mathrm{theoretical}\;\mathrm{drug}\;\mathrm{amount}}\;\times \;100$$

The drug concentration measured at each time point only reflects what is currently in the vials and ignores the drug removed in all prior samples, resulting in a lower calculated drug release than the actual amount released. Therefore, to correct this error, the cumulative drug amount in the above formula was calculated by adding the sum of all previously removed drug masses to the final measured amount using the formula below;

$$\mathrm{Actual}\;\mathrm{Qn}=\mathrm{Cn}\times \mathrm{Vt}+\sum_{i=1}^{n-1}\left(\mathrm{Ci}\times \mathrm{Vs}\right)$$

where

*Qn* = Cumulative mass of drug released at time point *n* (mg);
*Cn* = Measured concentration at the current time point *n* (mg/mL);
*Vt* = Total volume of the dissolution medium (mL);
Vs = Volume of the sample withdrawn (mL);
Ci = Measured concentration at previous time points (from *i* = 1 to *n* − 1).

### 2.7. Bacterial Strain Preparation

*S. aureus* strains ATCC 29213, ATCC 43300 (MRSA), and ATCC BAA-977, and *P. aeruginosa* strains ATCC 27853 and MDR 7067 were cultured on Mueller–Hinton agar (MHA) overnight at 37 °C. All bacterial suspensions were adjusted to a turbidity equivalent to that of a 0.5 McFarland standard.

#### 2.7.1. Determination of Minimum Inhibitory Concentration (MIC)

To determine MIC of AgNPs, BST, or AgNPs + BST, two-fold serial dilutions of them were prepared to obtain 500, 250, 125, 62.5, 31.25, 15.6, 7.8, and 3.9 μg/mL range of concentrations. One hundred microliters of either of diluted components were mixed with 100 µL of MHB containing bacterial suspensions and added to a 96-well plate. The bacterial inoculum concentration was maintained at 1 × 10^6^ colony-forming units/mL (CFU/mL). The cultures were incubated at 37 °C overnight with continuous shaking at 160 rpm. Absorbance was measured at 600 nm using a microplate reader. The minimum concentration at which there was no bacterial growth (i.e., lack of turbidity) was the MIC of AgNPs, BST, or AgNPs + BST.

#### 2.7.2. Evaluation of Zone of Inhibition

The zone of inhibition assay for the AgNP/BST-loaded fibers was performed using the agar diffusion method. AgNPs and AgNPs + BST, which exhibited antibacterial activity in the MIC test, were evaluated against the studied bacterial strains. MHA plates were incubated with 100 μL of 1.5 × 10^8^ CFU/mL of *P. aeruginosa* ATCC 27853, *S. aureus* ATCC 29213, *S. aureus* MRSA ATCC 43300, *P. aeruginosa* MDR 7067, or *S. aureus* ATCC BAA-977. Electrospun AgNP/BST-loaded PVA/CS fibers weighing 1 mg, which contains 3.7 μg/mg each of AgNPs and BST, and 1 mg blank PVA/CS fibers were placed over MHA, while 20 μL of diluted AgNPs at 3.7 μg concentration were added to sterile disks. MHA plates were incubated overnight at 37 °C, and the diameters of the inhibited zones were measured in millimeters (mm) using a digital micrometer.

#### 2.7.3. In Vitro Cytotoxic Effects

The cytotoxicity of AgNPs, BST, and AgNPs + BST was assessed by MTS assay using HFF1 cells. Serial dilutions of the compounds were prepared from 3.9 to 500 µg/mL. Cells were seeded in DMEM complete media at a density of 1.5 × 10^4^ cells per well in 96-well plates and kept in an incubator overnight at 37 °C and 5% CO_2_ in a humidified atmosphere. Following cell attachment, the medium was replaced with fresh medium containing AgNPs, BST, or AgNPs + BST at specified concentrations and the plates were further incubated for 24, 48, or 72 h. Cell viability at each time point was assessed using the MTS assay according to the manufacturer’s instructions. Absorbance was measured at 490 nm using a microplate reader (BIOTEK Instruments Inc., Winooski, VT, USA) and cell viability was measured according to the following equation:

Cell Viability % = (S − T)/(H − T) × 100

where S = absorbance of cells treated with the test compound, T = absorbance of cells treated with Triton X-100, and H = absorbance of untreated cells.

### 2.8. Statistical Analysis

All experiments were conducted with at least three independent replicates; the results were analyzed, plotted, and expressed as mean ± SD. The fiber diameters, DL, EE%, drug release, and regression equation of the HPLC analysis (mean and SD) were analyzed using Microsoft Excel. OriginPro^®^ 2025 (OriginLab Corporation, Northampton, MA, USA) software was used to plot the size distribution of the nanofibers, FTIR, XRD, and drug release profile figures. In vitro, the MIC was analyzed using one-way ANOVA per strain and group (positive control + 8 concentrations) followed by Dunnett’s multiple-comparison test of every concentration against the positive control. The Cumulative Release % was analyzed using a two-tailed Welch’s *t*-test comparing AgNPs vs. β-sitosterol at each time point, and the ZOI was analyzed using Tukey’s HSD post hoc test to compare the three groups within each strain. The nanofiber diagram was measured with ImageJ 1.54 g (Wayne Rasband and contributors, National Institutes of Health (Bethesda, MD, USA). *p* < 0.05 was considered to be statistically significant.

## 3. Results

### 3.1. Morphological Characterization of Loaded Fibers

**Figure 1:**
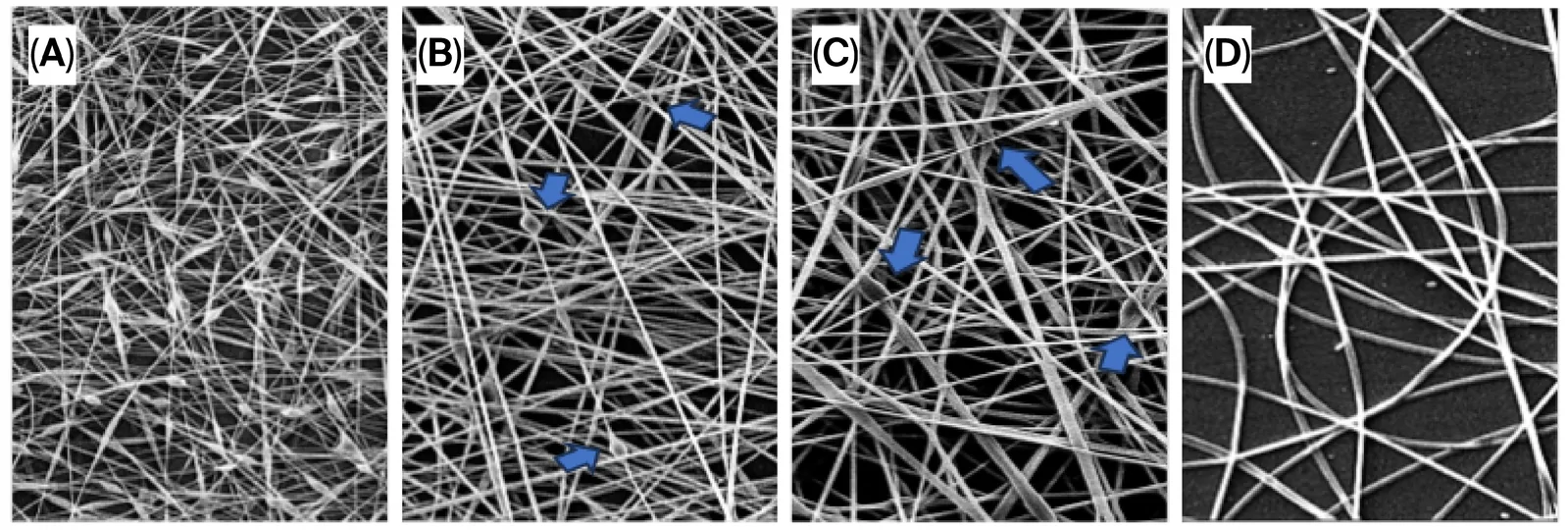
SEM of fiber structure for four different ratios for PVA/CS: (**A**) 50:50, (**B**) 60:40, (**C**) 70:30, and (**D**) 80:20. Arrows indicate the elongated, and ultrafine fibers with fewer beads.

Figure 1 shows the SEM micrographs of the morphology of AgNPs/BST-loaded nanofibers fabricated at different PVA/CS ratios (*v*/*v*). Drastic morphological changes were observed at 50:50 ratio of PVA/CS where nanofibers displayed numerous beads Figure 1A), which could be attributed to higher CS percentage of the polymer solution. By altering PVA/CS ratios to 60:40 and 70:30, fibers gradually elongated, and ultrafine fibers with fewer beads were formed (Figure 1B,C). Setting PVA/CS ratio to 80:20 led to complete formation of electrospun fibers with normal diameters (Figure 1D). Additionally, these fibers were smooth in texture and easier to peel from the foil without any breakage or loss of material. Although the PVA/chitosan ratios played a crucial role determining the nanofiber structure, other processing conditions such as voltage, needle diameter, needle tip-to-collector distance and the flow rate of spinning solution also influence the fiber morphology.

### 3.2. Morphological Assessment of Loaded Nanofibers

**Figure 2:**
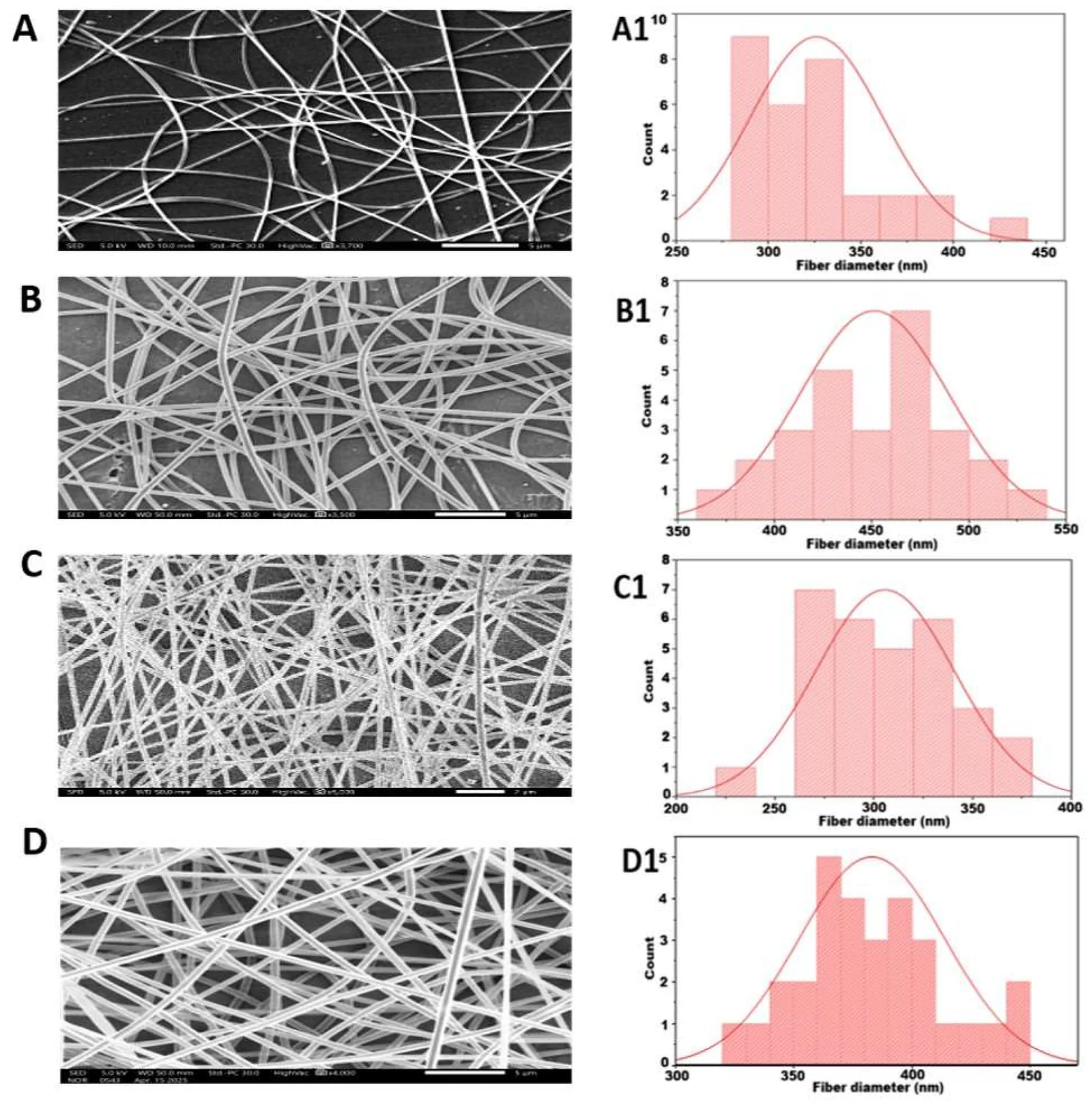
SEM images showing the morphology of the different nanofibers: (**A**) PVA/CS, (**B**) PVA/CS/BST, (**C**) PVA/CS/AgNPs, and (**D**) PVA/CS/BST/AgNPs. On the right, bar graphs of the diameter distributions of (**A1**) PVA/CS, (**B1**) PVA/CS/BST, (**C1**) PVA/CS/AgNPs, and (**D1**) PVA/CS/BST/AgNPs. The columns in the right panel indicate the physical measurements of the nanofiber diameters grouped into specific size intervals and superimposed curves represent Gaussian distribution curves.

The SEM images in Figure 2A–D show the morphology of the PVA/CS, PVA/CS/BST, PVA/CS/AgNPs, and PVA/CS/BST-AgNPs nanofibers respectively as smooth, non-beaded, and non-porous surfaces. The bar graphs (Figure 2A1–D1) of PVA/CS, PVA/CS/BST, PVA/CS/AgNPs, and PVA/CS/BST-AgNPs nanofibers indicate average diameters of the nanofibers in nanometers. The SEM observations and fiber diameter distributions clearly show the influence of compositional changes on PVA/CS nanofibers. The PVA/CS fibers had smooth surfaces with almost no bead formation (Figure 2A), showing that the electrospinning conditions were stable and that PVA/CS were compatible. The morphology of these fibers is uniform, as the fiber diameter distribution was relatively narrow suggesting that the jet stretching was consistent throughout the spinning process. The average PVA/CS nanofiber diameter was found to be 325.9 ± 35.8 nm (Figure 2A1). Following the addition of BST to the mix, the fiber appearance did not vary but its thickness increased (Figure 2B) This is probably due to the change in the thickness of the solution thereby altering interactions between the polymer and BST, making it harder for the fibers to stretch during electrospinning and consequently affecting the fibers’ appearance. The average diameter of PVA/CS/BST nanofibers was 451.2 ± 37.4 nm (Figure 2B1). These fibers were found to be packed tightly together, indicating that the silver nanoparticles improved the solution’s conductivity and promoted the fiber formation (Figure 2C). The average diameter of the PVA/CS/AgNPs nanofibers was noted to be 305.5 ± 35.2 nm (Figure 2C1). The PVA/CS/BST/AgNPs system formed a well-organized, uniform mat with a very regular structure (Figure 2D), effectively producing high-quality fibers with a diameter of 383 ± 30.8 nm (Figure 2D1). The diameters of different nanofibers are presented in Table 2.

**Figure 3:**
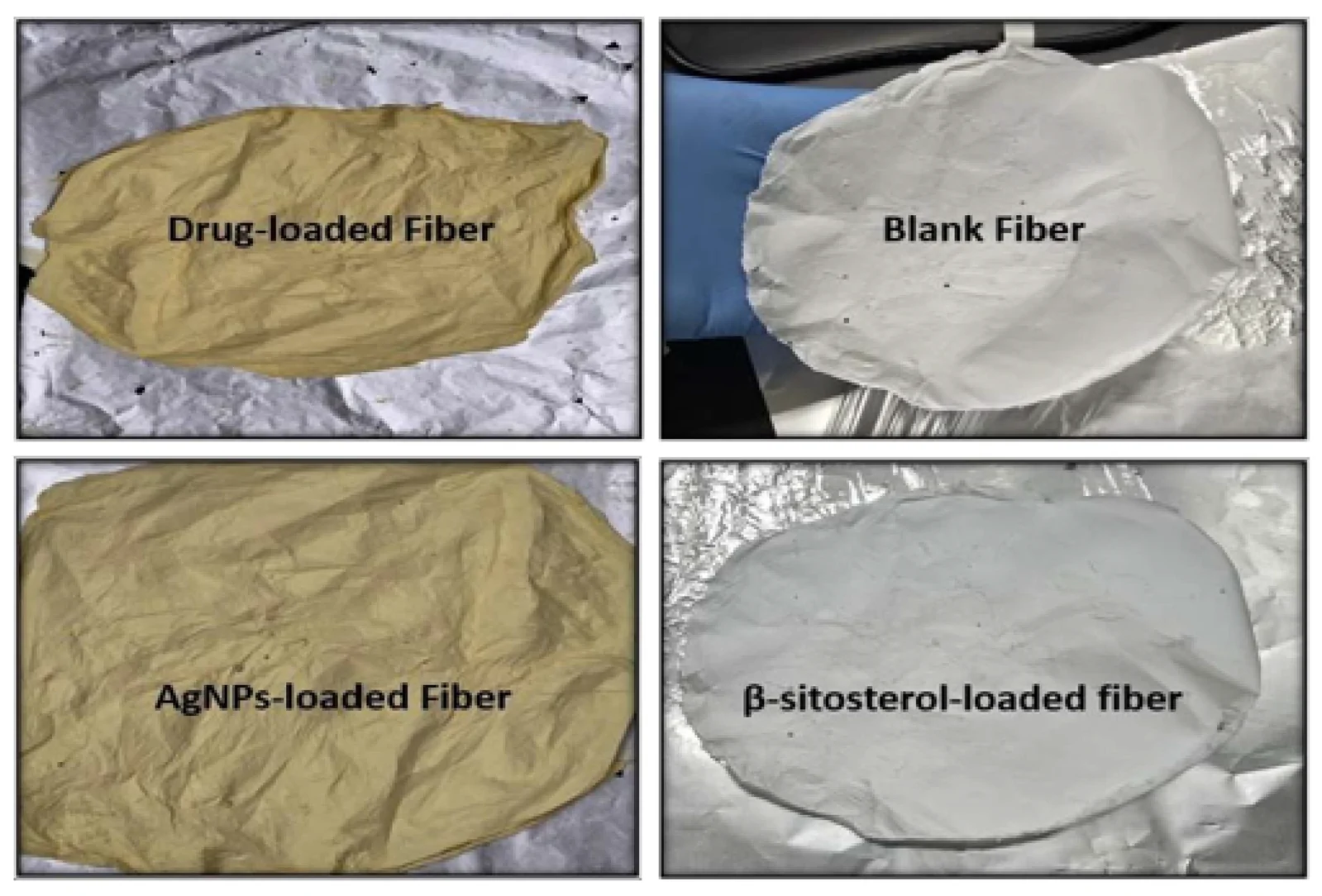
Digital images of the electrospun fiber after fabrication. The images show the drug-loaded, blank, AgNP-loaded, and BST-loaded fiber formulations. A yellow color was observed for the fiber, indicating the presence of AgNPs.

Figure 3 shows photographs of fabricated fiber mats. A visual inspection of the prepared formulations shows distinct differences in appearance and color: the blank fiber and BST-loaded fiber mats had white, opaque, and smooth surfaces, whereas the AgNP-loaded fiber and the drug-loaded fiber with both BST and AgNPs were yellow in color due to the presence of AgNP solution. All fiber mats formed continuous free-standing membranes that could be removed intact from the collector (foil) without any visible tearing. The prepared fibers were characterized by homogeneous macroscopic structures, and no obvious cracks, holes, or phase separation were observed by naked eye.

**Figure 4:**
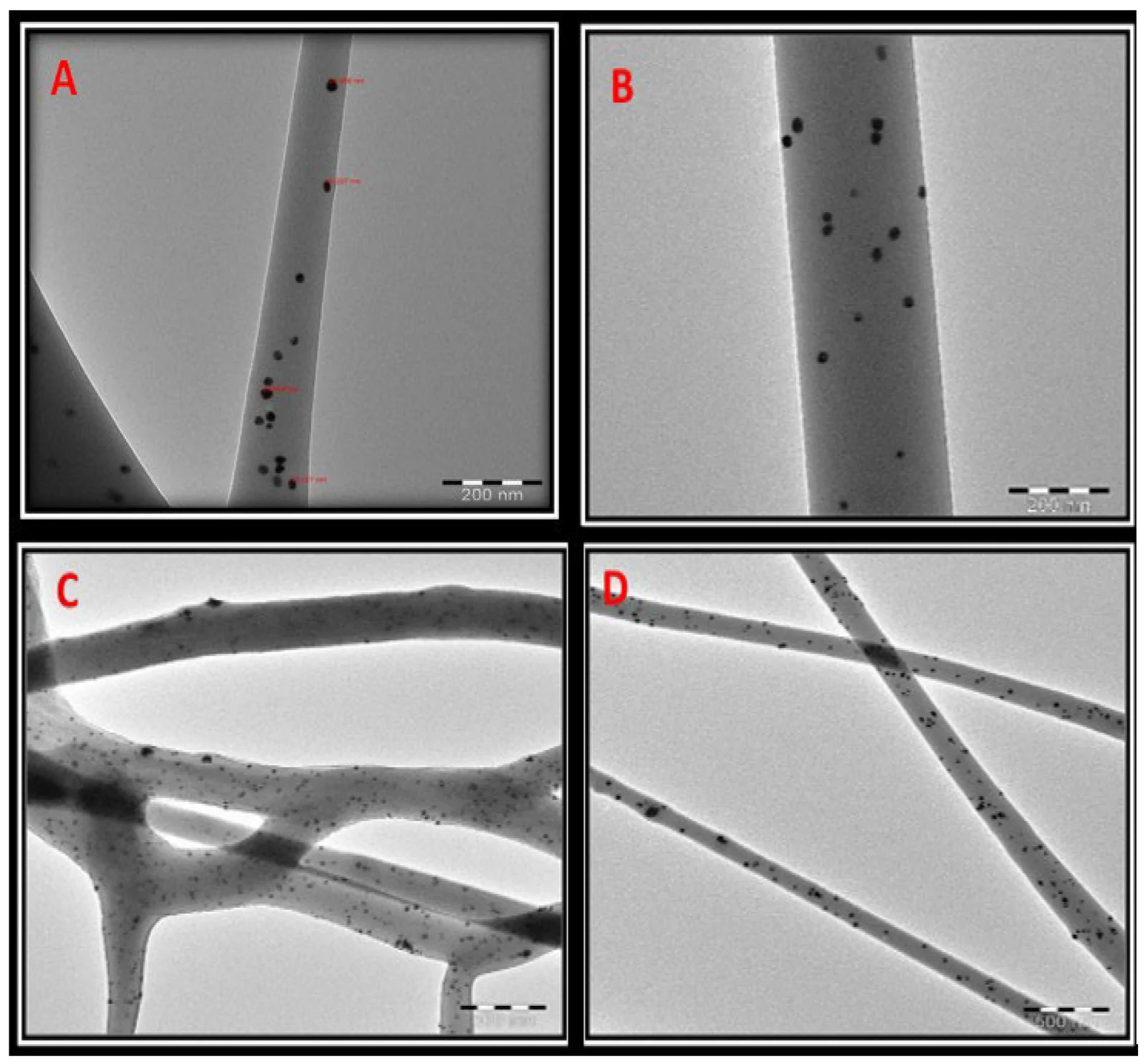
TEM images of nanofibers, showing the distribution of silver nanoparticles within the electrospun fibers. (**A**,**C**)—PVA/CS/BST/AgNPs 200–500 nm; (**B**,**D**)—PVA/CS/AgNPs 200–500 nm.

The TEM images show the successful formation of PVA/CS/BST/AgNPs (Figure 4A,C) and PVA/CS/AgNPs nanofibers (Figure 4B,D). The average diameter AgNP containing fibers was found to be approximately 15 nm and the images show that the AgNPs were loaded into the nanofiber and distributed throughout the fiber.

### 3.3. FTIR Analysis

**Figure 5:**
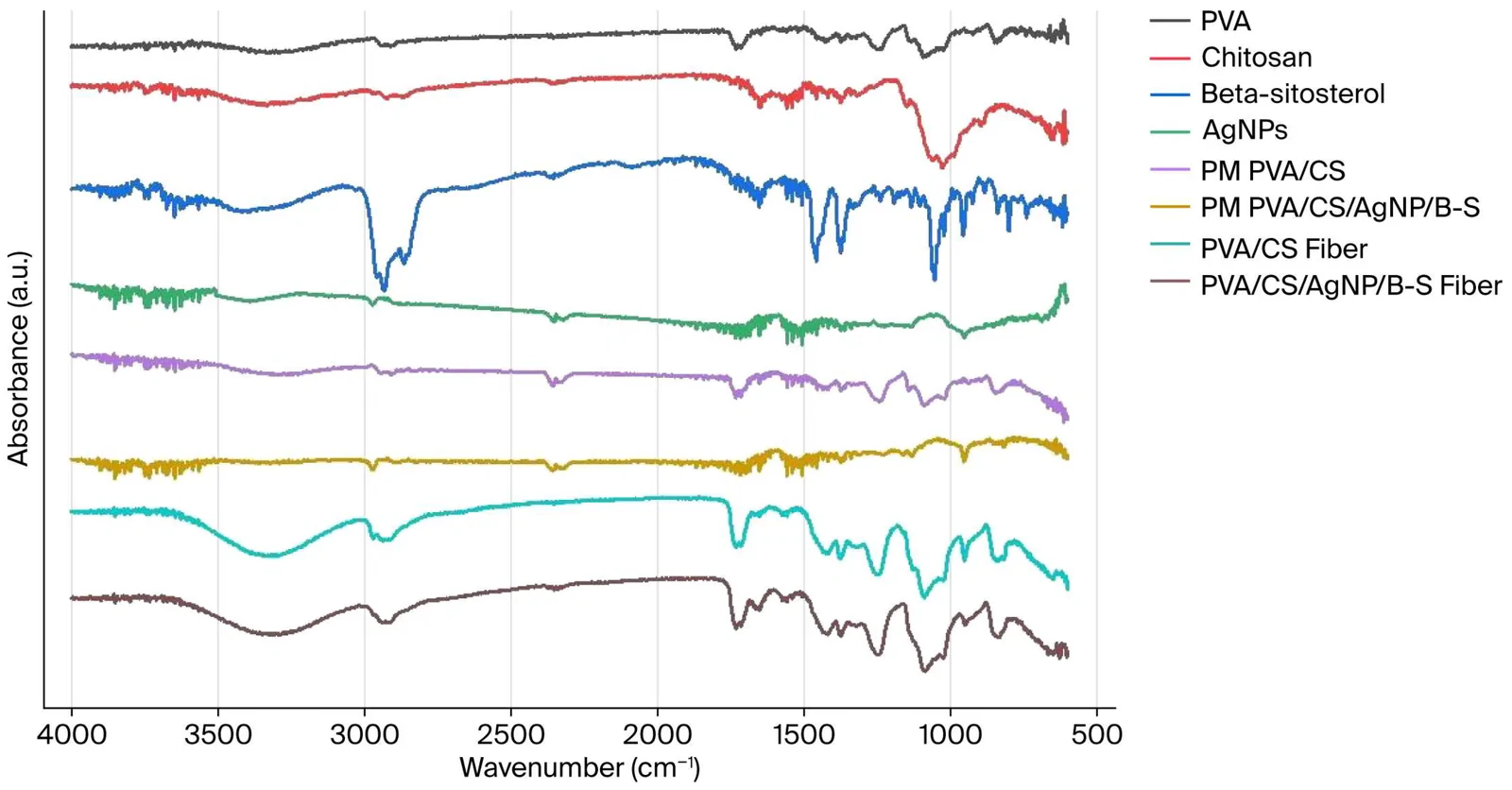
FTIR spectrum of PVA, CS, BST, AgNPs, PM PVA/CS, PM PVA/CS/BST/AgNPs, Blank, and AgNPs and BST-loaded fiber.

The FTIR was conducted to identify the functional groups involved in the reduction and stabilization of PVA, CS, BST, AgNPs, a physical mixture (PM) of PVA/CS, PVA/CS/BST/AgNPs, PVA/CS fiber, and PVA/CS/BST/AgNPs fiber, as shown in Figure 5. Pure PVA exhibited characteristic bands associated with O-H stretching around ~3300 cm^−1^, C-H stretching at ~2900 cm^−1^, and C-O-C vibrations in the 1080–1140 cm^−1^ region, consistent with its polymeric backbone. Chitosan showed a broad O-H/N-H stretching band centered near 3300–3400 cm^−1^, amide I and amide II bands at approximately 1650 and 1550 cm^−1^, respectively, and C-O stretching peaks around 1020–1080 cm^−1^. Beta-sitosterol exhibited distinctive bands corresponding to O-H stretching at ~3400 cm^−1^ and pronounced aliphatic stretch vibrations of CH_2_ group signals near 2850–2950 cm^−1^, in addition to CH_2_ bending and C-O stretching around 1460 and 1313 cm^−1^, respectively. Additionally, 1055 cm^−1^ (C-O-C) showed a clear displacement in absorbance. The AgNP band at 1340.31 cm^−1^ was associated with C-N stretching of aliphatic amines, while the peak at 1030.86 cm^−1^ corresponded to C-O stretching vibrations of alcohols. Peaks between 864.95 cm^−1^ and 825.81 cm^−1^ were indicative of C-H bending in alkenes and aromatic moieties. Comparative FTIR fingerprint details are presented in Table 3.

### 3.4. X-Ray Diffraction (XRD) Assessment

**Figure 6:**
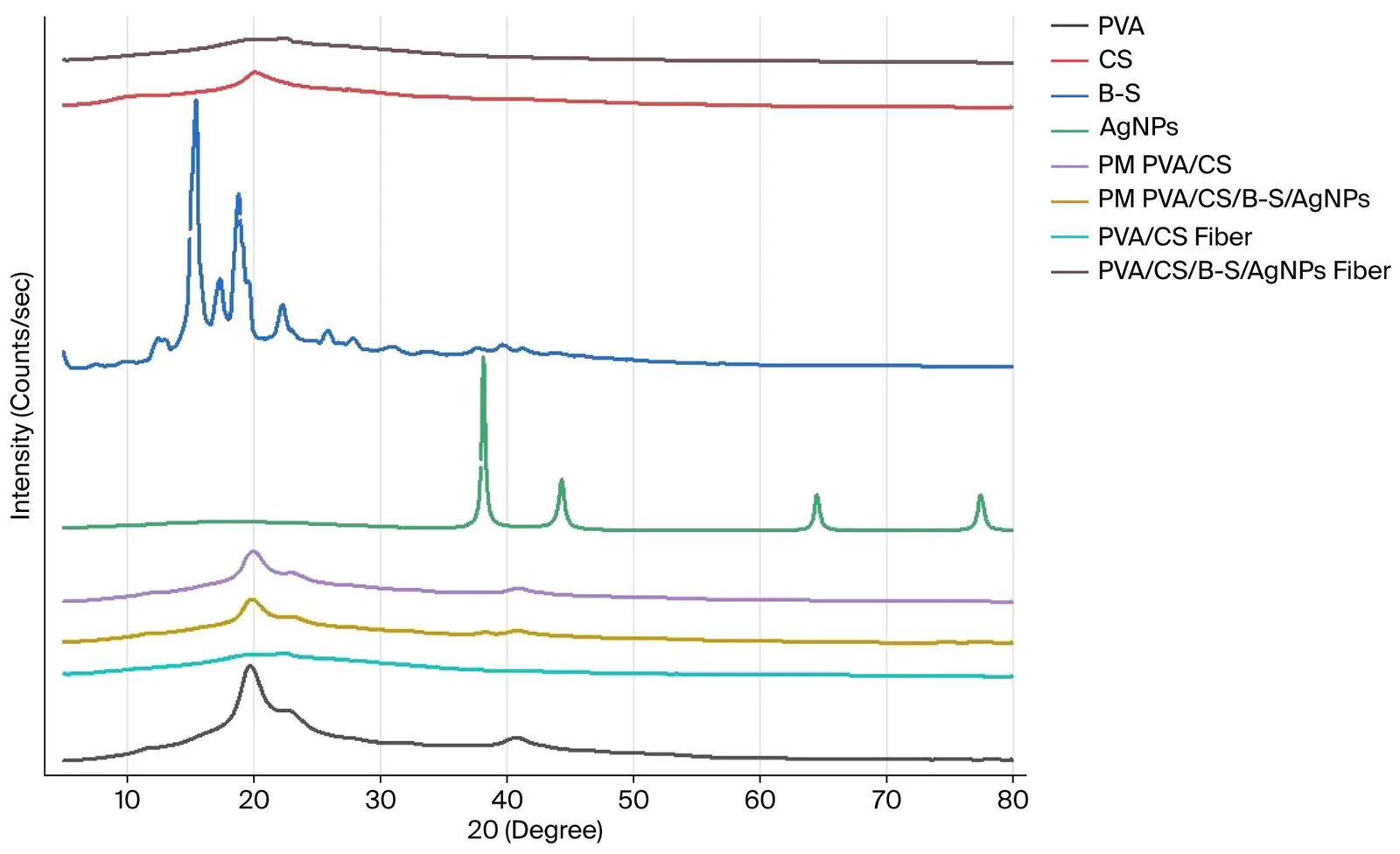
XRD patterns of PVA, CS, BST, AgNPs, PM PVA/CS, PM PVA/CS/BST/AgNPs, blank, and drug-loaded nanofibers. Abbreviations: PVA, polyvinyl alcohol; CS, chitosan; BST, β-sitosterol; AgNPs, silver nanoparticles; PM, physical mixture.

The data of XRD analysis are shown in Figure 6. XRD was used to determine crystallinity to assess the amorphous state of the drug, materials, and nanofibrous membranes. PVA showed a broad diffraction peak around 2θ = 20°, which is typical of its semi-crystalline structure [35]. In contrast, chitosan displayed a weak, broad diffraction peak at 2θ around 20 and 10 [36], indicating its mostly amorphous structure. BST showed several sharp, strong diffraction peaks, confirming its highly crystalline structure [37]. The diffraction pattern of silver nanoparticles showed clear peaks at about 2θ = 38°, 44°, and 64°.

Following blending and electrospinning, a marked decrease in peak intensity and considerable peak broadening were observed in the PVA/CS blend and the drug-loaded nanofibrous membranes, indicating reduced overall crystallinity. There was also an absence or attenuation of the distinctive crystalline peaks of BST and AgNPs in the composite nanofibers (Figure 6).

### 3.5. Drug Loading (DL) and Encapsulation Efficiency Percentage (EE%) of AgNPs

**Figure 7:**
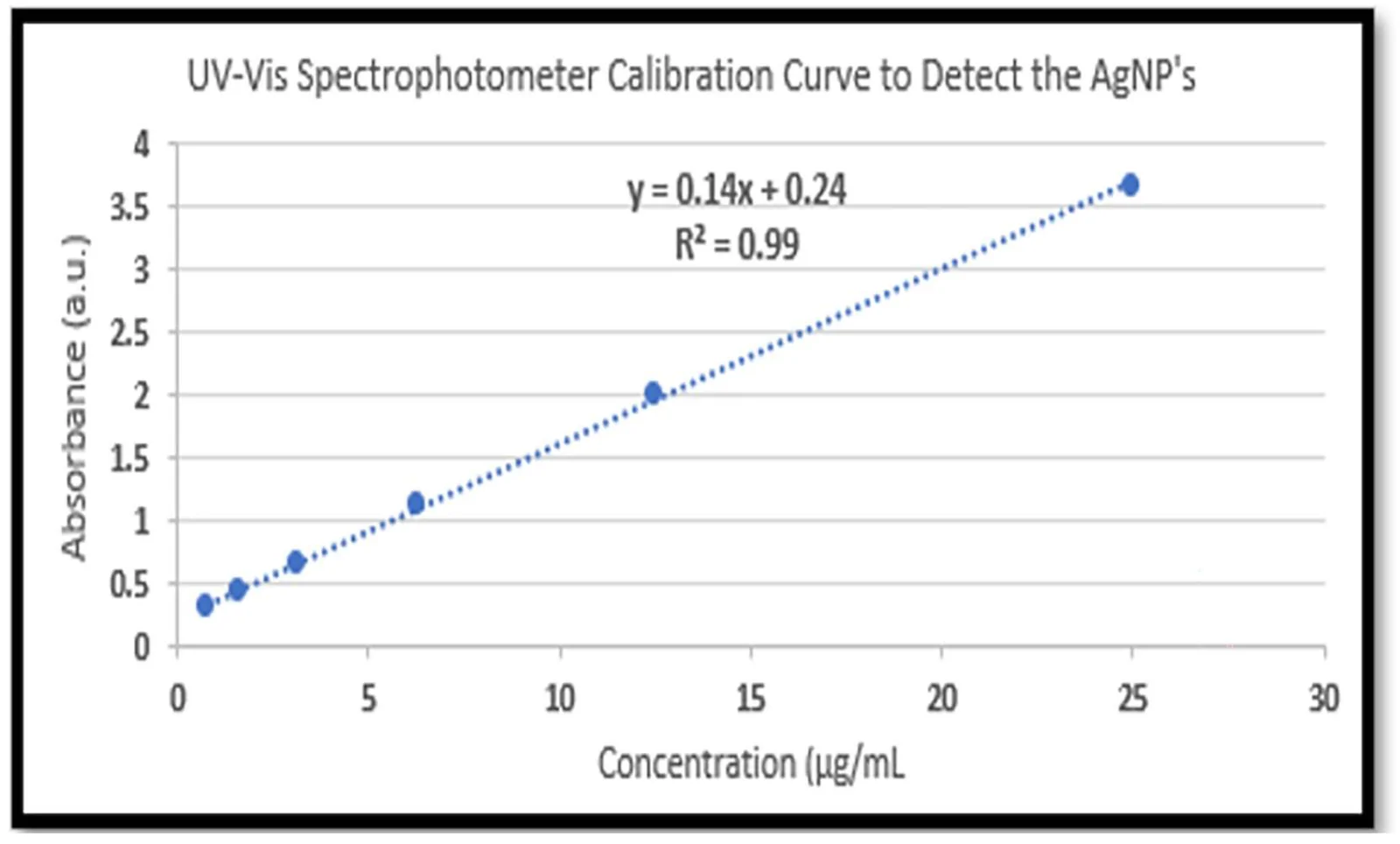
UV–Vis spectrophotometer calibration curve for detecting the AgNP concentration, at concentrations of 0.7, 1.56, 3.125, 6.25, 12.5, and 25 µg/mL.

**Figure 8:**
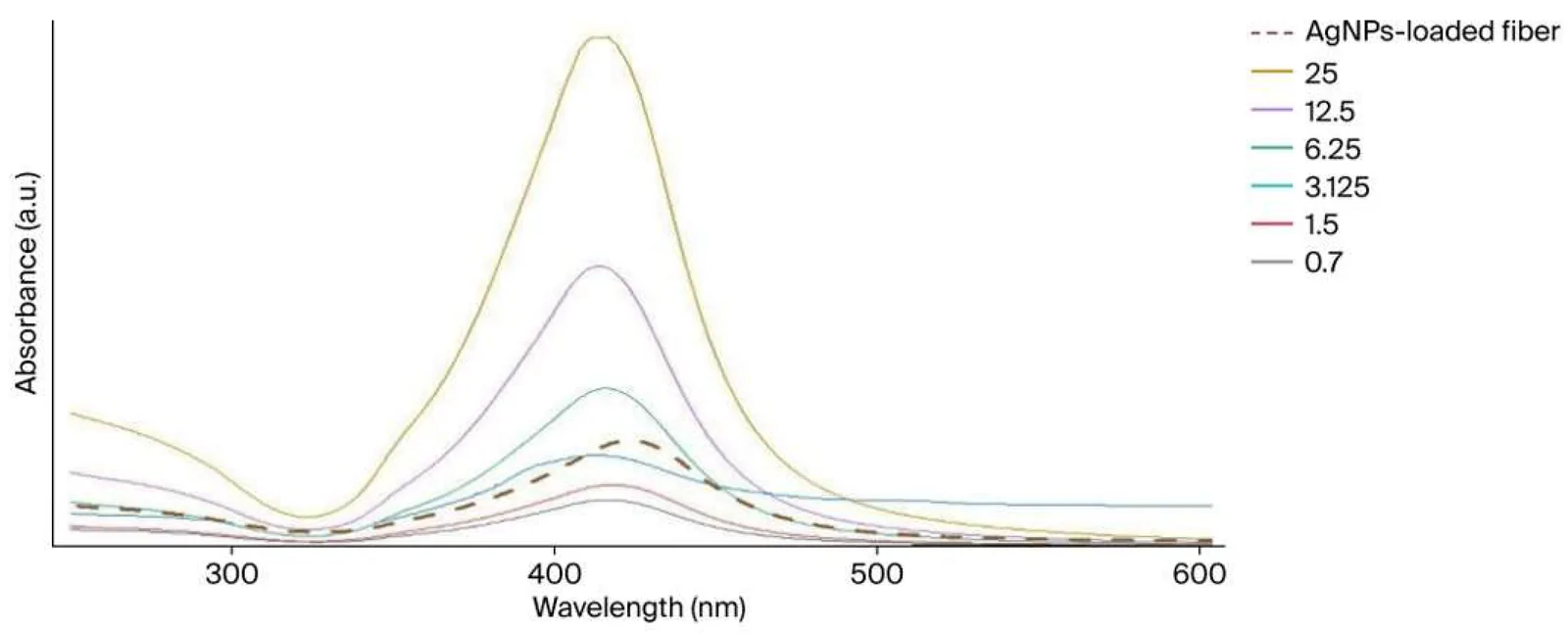
UV–Vis spectrophotometer standard curve (0.7, 1.56, 3.125, 6.25, 12.5, and 25 µg/mL); the dashed line of the drug-loaded fiber sample shows the AgNP concentration.

A UV–Vis spectrophotometer was used to quantify AgNPs, and a calibration curve was obtained by plotting absorbance versus the concentration of silver nanoparticles (AgNPs) in the range of 0.7, 1.56, 3.125, 6.25, 12.5, and 25 µg/mL (Figure 7). The absorbance increased proportionally with increasing concentrations of AgNPs, with a strong linear relationship over the whole tested range. The regression equation obtained from linear regression analysis was *y* = 0.1388x + 0.2401, where y is the absorbance and x is the AgNP concentration (µg/mL). The coefficient of determination (R^2^ = 0.9995) demonstrates excellent linearity and confirms the reliability of the calibration model. The absorbance units for (0.7 µg/mL = 0.33), (1.56 µg/mL = 0.44), (3.125 µg/mL = 0.65), (6.25 µg/mL =1.13), (12.5 µg/mL = 2.0), and (25 µg/mL = 3.68) (Figure 8).

The AgNP loading, drug loading (DL), and encapsulation efficiency (EE) of the electrospun fibers were calculated using the calibration curve obtained from the UV–Vis analysis. Three batches collected from each fiber (nine samples) were analyzed and measured. Fiber 1 showed the highest AgNP content, with an average EE% of 87.4% and a DL of 3.77 µg/mg; Fiber 2 had the lowest values, with an average EE of 82% and a DL of 3.55 µg/mg; and Fiber 3 had intermediate values, with an average EE of 84.4% and a DL value of 3.6 µg/mg (Table 4). The encapsulation efficiency of AgNP-loaded fibers was 84.6% overall, and the average drug loading was 3.64 µg/mg fiber. The results for the three fiber samples were similar, indicating good reproducibility of the loading procedure.

### 3.6. Drug Loading (DL) and Encapsulation Efficiency Percentage (EE %) Determination of BST by HPLC

**Figure 9:**
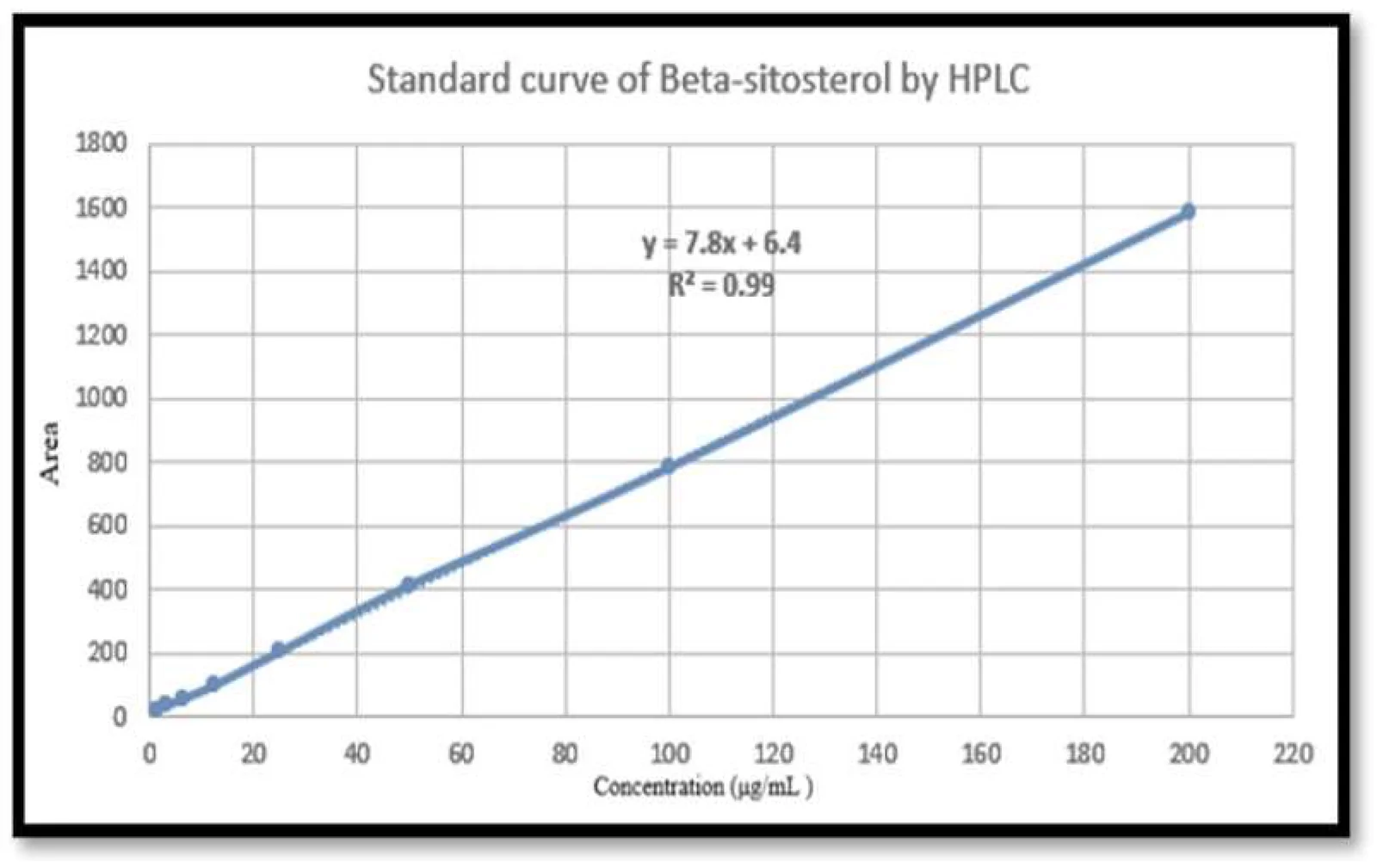
The standard curve from HPLC for different concentrations (1.5, 3.125, 6.25, 12.5, 25, 50, 100, and 200 µg/mL) of beta-sitosterol.

The BST standard curve was constructed using HPLC at concentrations of 1.5, 3.125, 6.25, 12.5, 25, 50, 100, and 200 µg/mL (Figure 9). All chromatograms showed a well-defined BST peak for all concentrations. The peak of BST was clearly detectable even at the lowest concentration evaluated (1.5 µg/mL), showing the good sensitivity of the HPLC method. At high concentrations (100 and 200 µg/mL), the chromatographic peaks were symmetric and well separated, confirming the applicability of the method for quantitative determination over a wide concentration range. The progressive increase in peak response with increasing concentration was then used to construct the BST calibration curve for the quantitative analysis of the samples (regression equation: y = 7.8636x + 6.4412, correlation coefficient (R^2^) = 0.9998). EE% and DL of the beta-sitosterol-loaded fiber are shown in Table 5.

### 3.7. In Vitro Drug Release 

**Figure 10:**
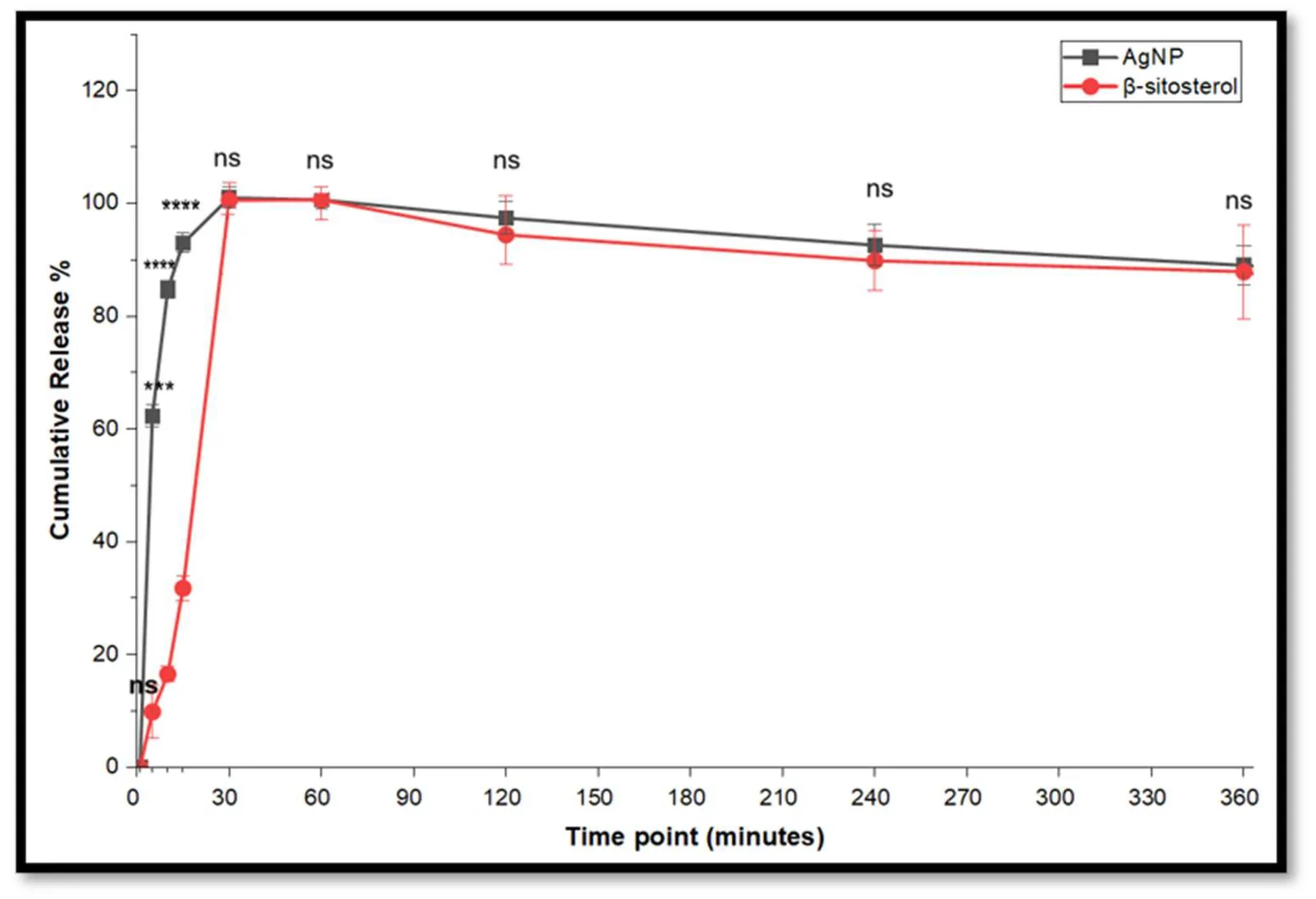
In vitro release profile of BST and AgNP from the fibers. Results are presented as mean ± SD of 3 independent experiments (n = 3). Two-tailed Welch’s *t*-test was used to compare the release of AgNPs and BST at each time point *** *p* < 0.001, **** *p* < 0.0001, ns = not significant).

The in vitro drug release data are presented in Figure 10. The loaded nanofibers showed a rapid burst release of AgNP from 0 to 30 min, where a 60% release of AgNPs (was recorded after 5 min followed by its complete release after 30 min). Conversely, BST demonstrated a 15% release after the initial 5 min followed by increases of 33% and 100% releases after 15 and 30 min respectively. AgNPs and BST releases approached completion within 30 min and remained approximately constant up to 360 min.

### 3.8. Antibacterial Minimum Inhibitory Concentration (MIC)

**Figure 11:**
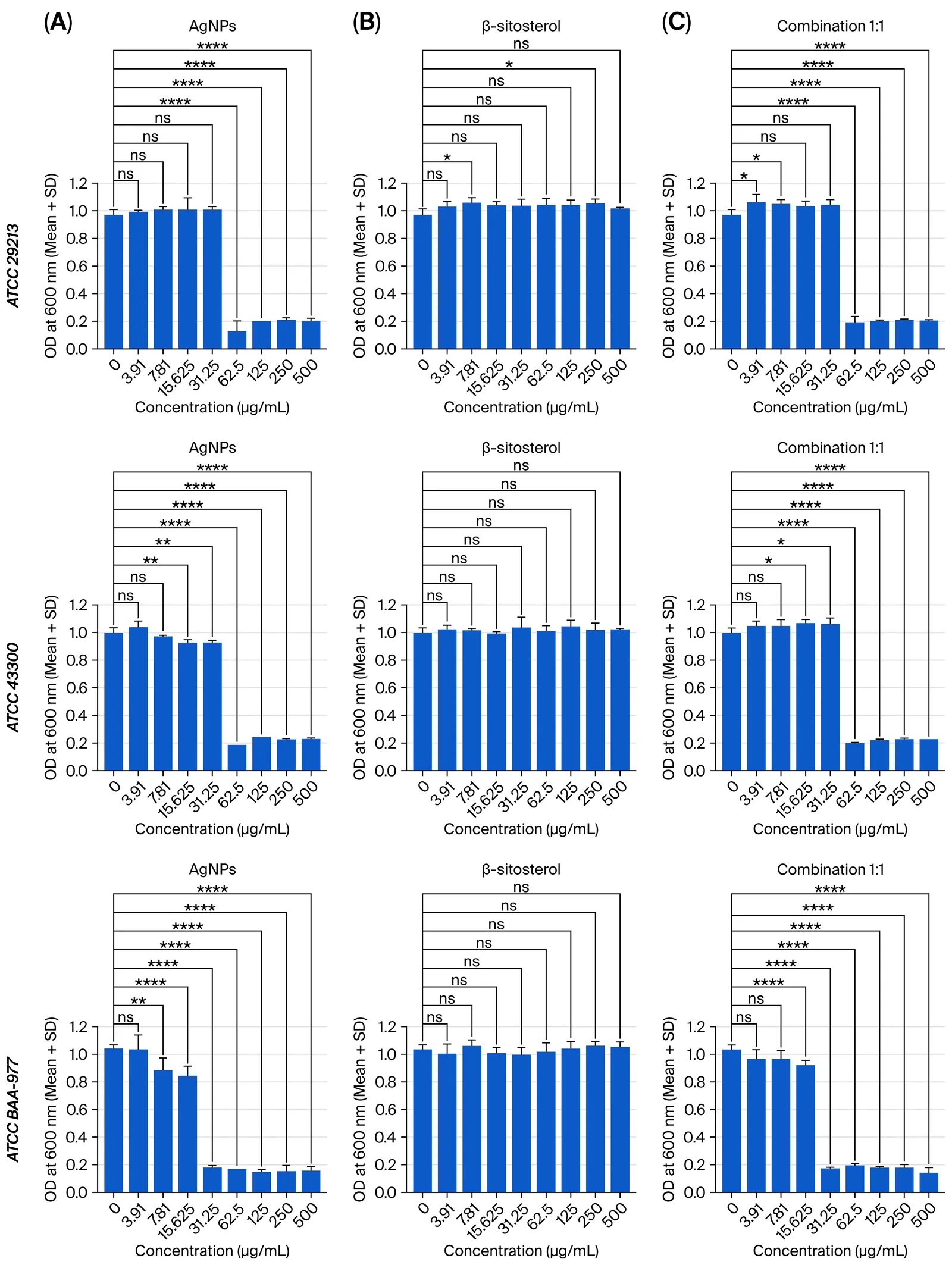

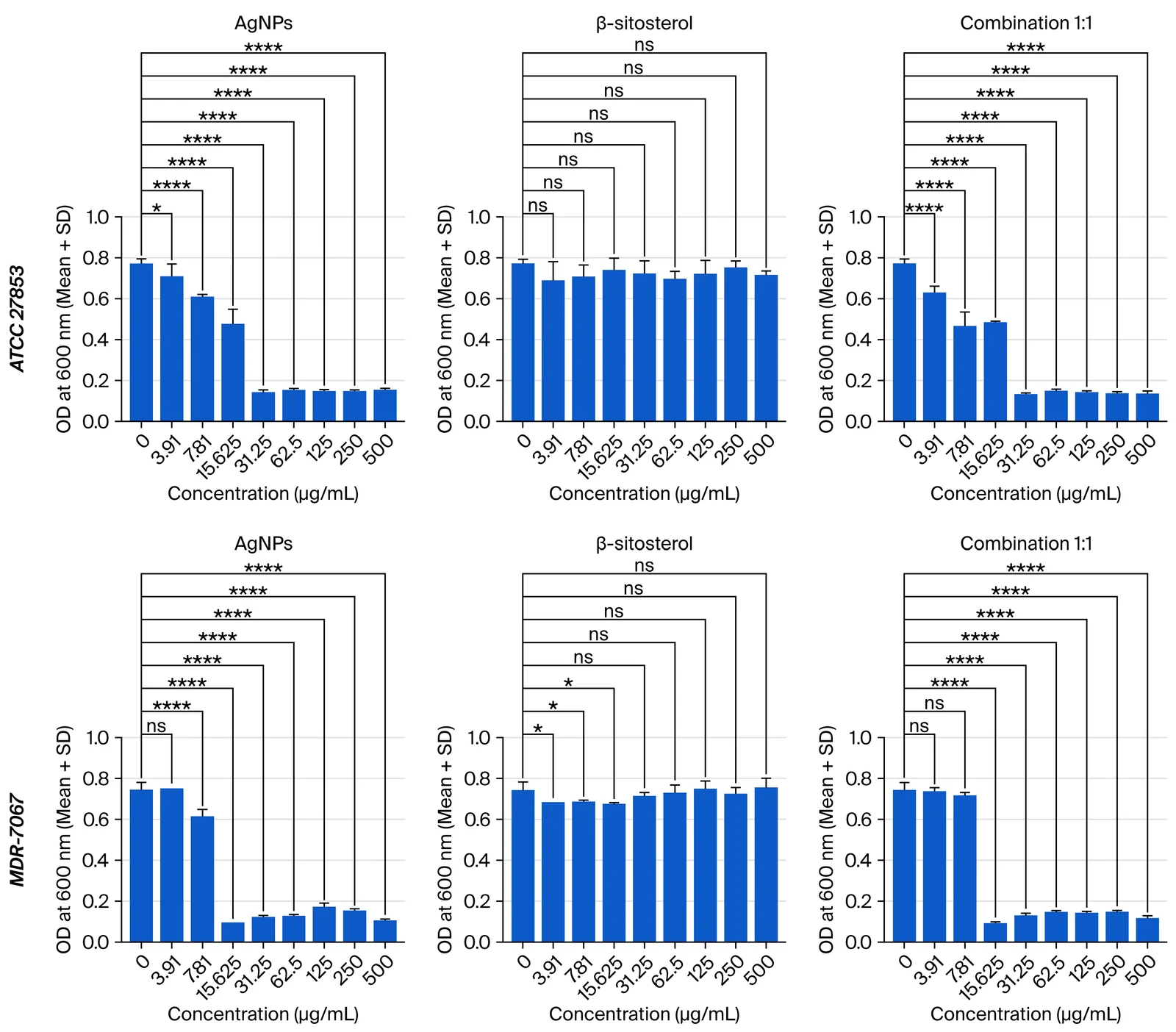
Effect of AgNPs (**A**), BST (**B**), and combination 1:1 (**C**) against three different strains of *Staphylococcus aureus* and two different strains of *P. aeruginosa* at dilution concentrations of 500 to 3.9 µg/mL on the growth (OD at 600 nm) of ATCC 29213, ATCC 43300, ATCC BAA-977, ATCC 27853, and MDR 7067. All experiments were performed in triplicate, and results are presented as mean + SD of 3 independent experiments (n = 3). Statistical analysis was performed using one-way ANOVA followed by Dunnett’s multiple-comparison test against control (* *p* < 0.05, ** *p* < 0.01, **** *p* < 0.0001; ns = not significant).

Growth inhibitory effects of AgNPs, BST and AgNP + BST against the studied bacterial strains are presented in Figure 11. As the MIC values suggest, AgNPs and the combination of AgNPs with BST were effective in the growth inhibition of ATCC 29213 and ATCC 43300 strains of staphylococcus aureus at 62.5 µg/mL and up to 500 µg/mL, whereas the MIC of AgNPs and AgNPs + BST was found to be 31.2 µg/mL against ATCC BAA-977. Contrarily, BST alone had no antibacterial effect against all the three types of studied *Staphylococcus aureus* strains even up to a 500 µg/mL dose. Against the ATCC 27853 strain of *Pseudomonas aeruginosa*, the MICs of AgNPs and the combination of AgNPs and BST were noted to be 31. 2 µg/mL and higher, and against MDR-7067 strain the MICs of AgNPs and AgNPs + BST were 15.6 µg/mL Conversely, BST did not show any growth inhibitory effect against the tested strains of *Pseudomonas aeruginosa.* All experiments were performed in three replicates, and data are presented as mean ± standard deviation of three independent experiments.

### 3.9. Zone of Inhibition 

**Figure 12:**
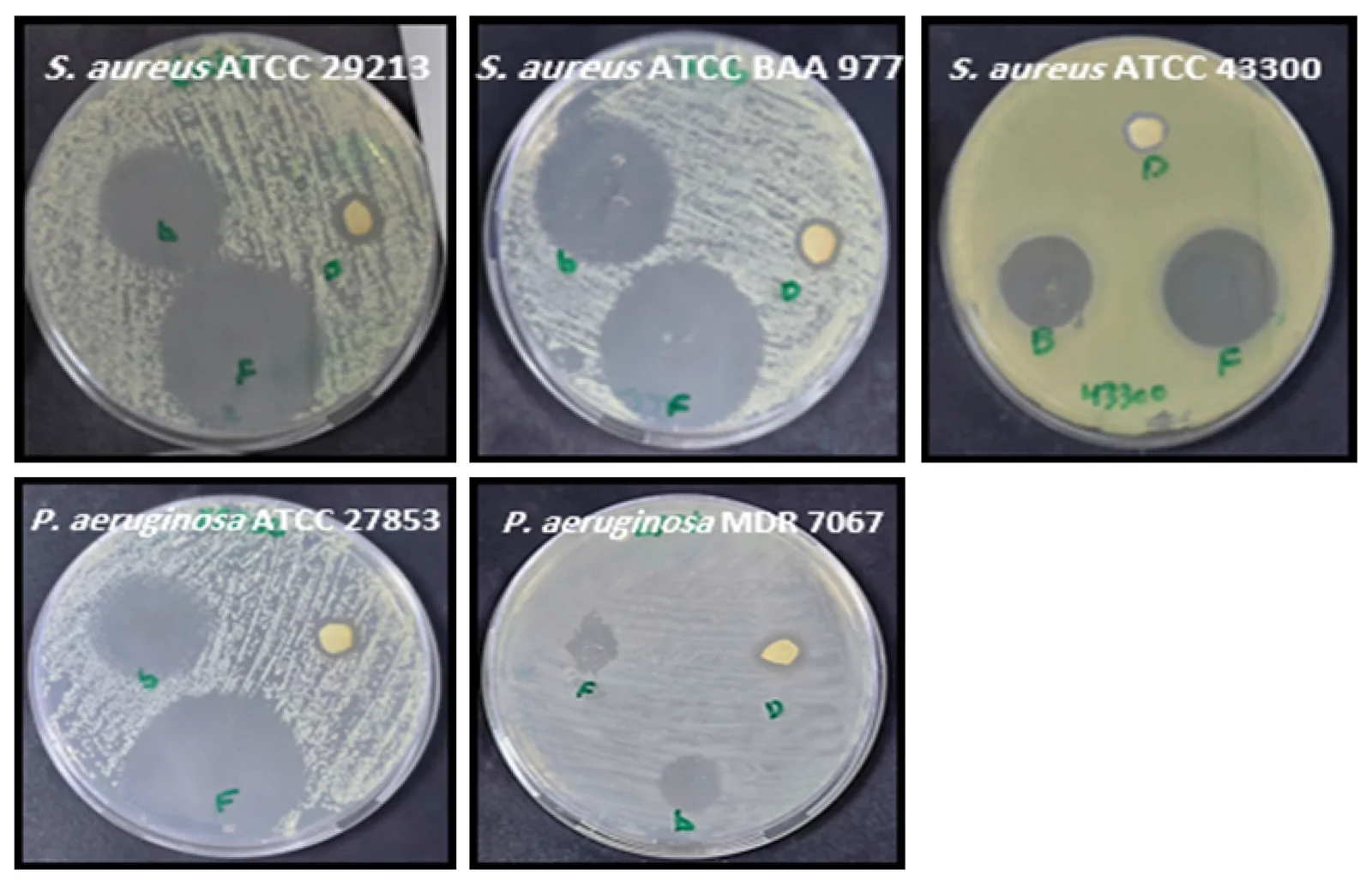
The antibacterial activity of AgNP disks (D), blank fibers (PVA/chitosan only) (B), and AgNPs and BST-loaded PVA/chitosan fibers (F) against *S. aureus* ATCC 29213, BAA 977, and ATCC 43300 strains and against *P. aeruginosa* ATCC 27853, and *P. aeruginosa* ATCC 7067 strains were tested using the agar diffusion method. The drug-loaded fibers showed significantly higher ZOI values against all the tested bacteria except MDR7067 strain of *P. aeruginosa* compared to AgNPs or blank nanofibers.

The antibacterial activities of the AgNPs and BST-loaded PVA/chitosan fibers were evaluated through the zone of inhibition test (also known as the disk diffusion test). Figure 12 shows representative zones of inhibition of studied bacterial strains, while the widths of the zones of inhibition are shown in Table 6.

**Figure 13:**
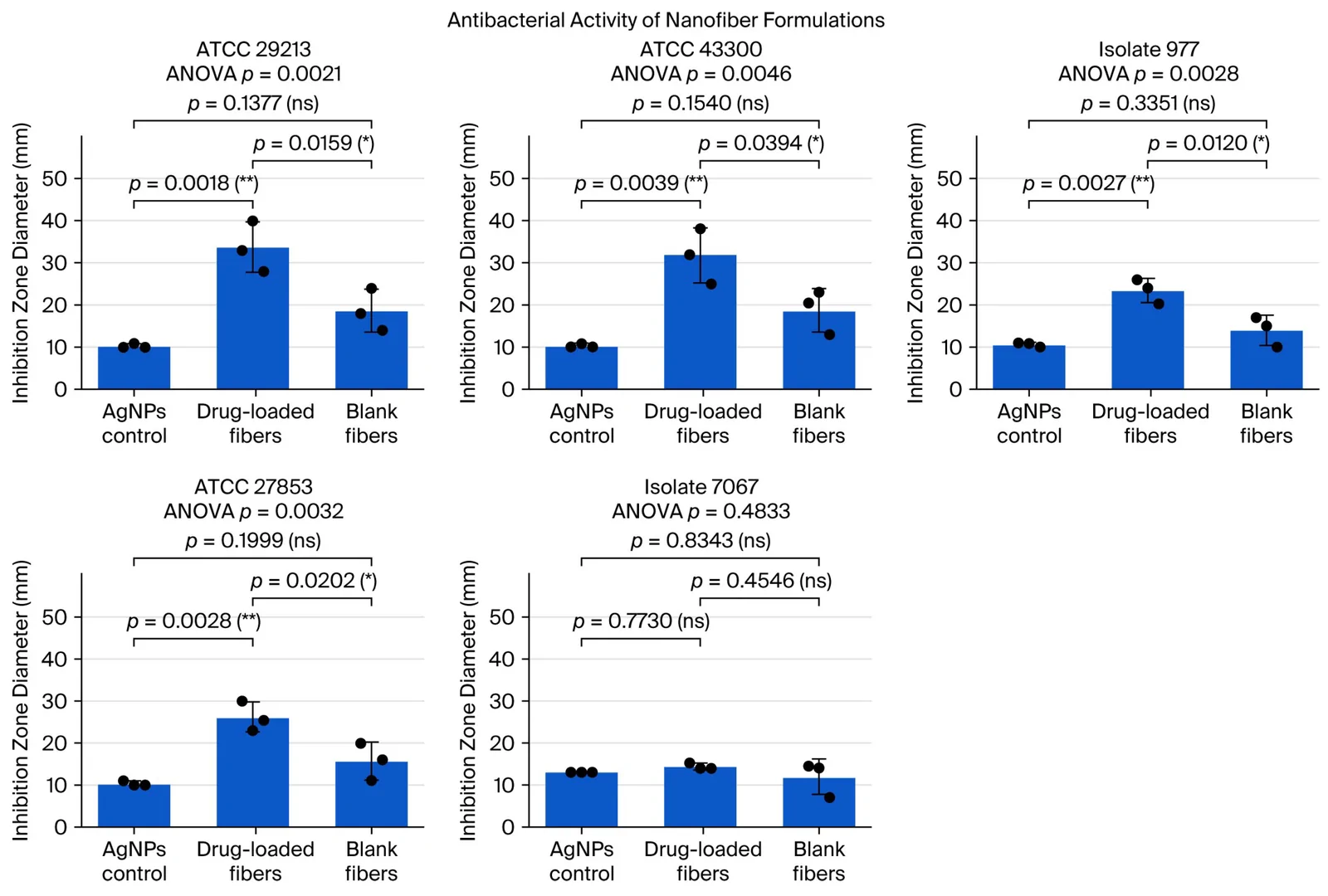
Antibacterial activities of AgNPs disk, AgNPs and BST-loaded PVA/chitosan nanofibers, and blank nanofibers (PVA/chitosan only). Results are presented as mean ± SD (n = 3). One-way ANOVA followed by Tukey’s HSD post hoc test was used to compare the three groups within each strain. * *p* < 0.05; ** *p* < 0.01; ns = not significant. The data represent mean ± SD of 3 independent experiments (N = 3).

It was noted that the ZOI of AgNPs and BST-loaded fibers were significantly larger than that of AgNPs and blank fibers across the three strains of *S. aureus* and one strain of *P. aeruginosa* (Figure 13), while no significance was observed among AgNP’s disk, blank fibers and loaded fibers. The ZOI of AgNPs and that of blank fibers were comparable among all the five tested strains (Figure 13). The differences in ZOI between AgNPs and loaded nanofibers were more significant than those between loaded nanofibers and blank fibers across three strains of *S. aureus* and one strain of *P. aeruginosa*.

### 3.10. In Vitro Cell Viability Assessment

**Figure 14:**
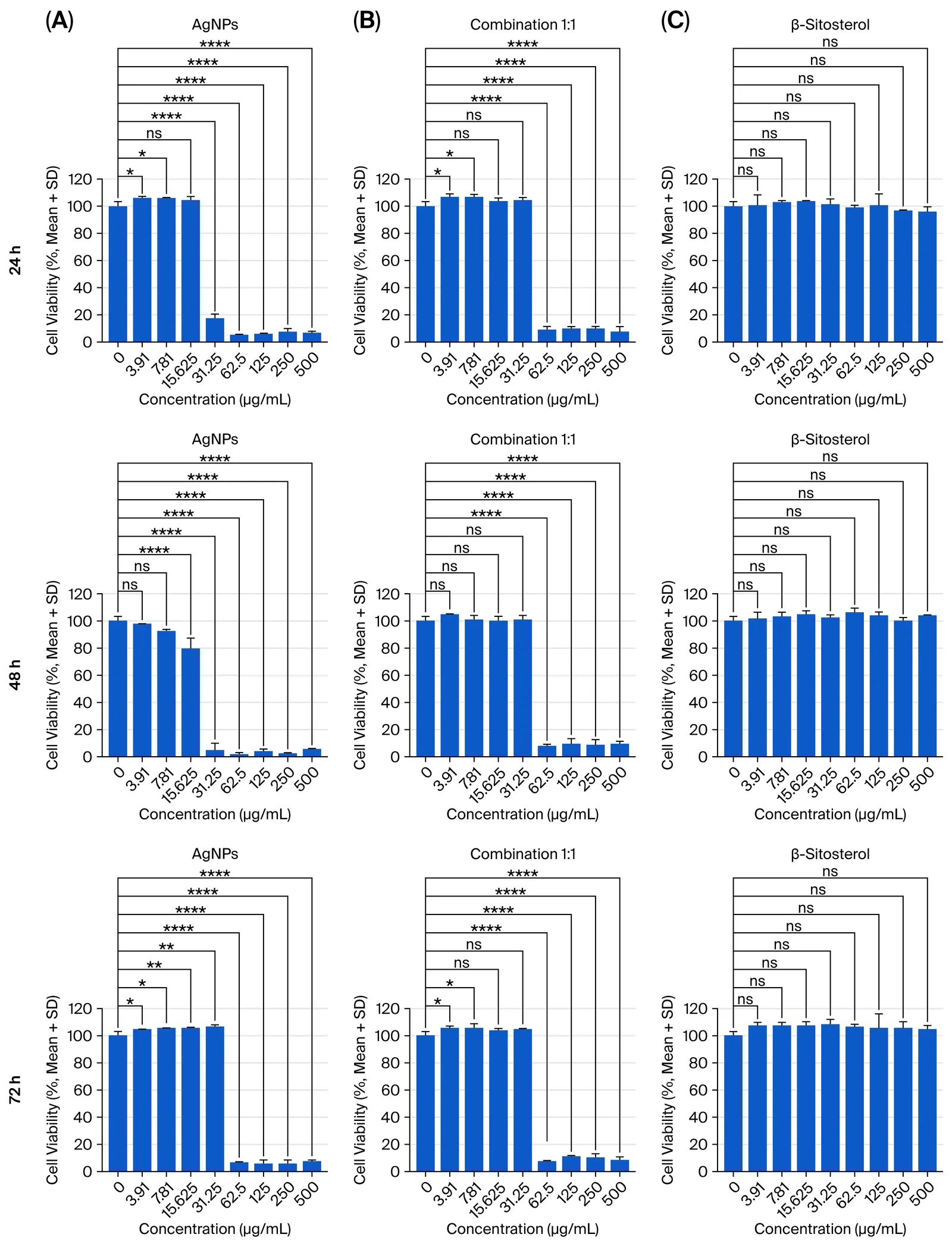
Cell viability in response to (**A**) AgNPs, (**B**) AgNPs + BST, and (**C**) BST exposure of HFF1 cells for 24 h, 48 h, or 72 h. Statistical analysis was performed using one-way ANOVA followed by Dunnett’s multiple-comparison test against the positive control (* *p* < 0.05, ** *p* < 0.01, **** *p* < 0.0001, ns = not significant). All experiments are performed in triplicates and data are presented as mean ± SD of 3 independent experiments (n = 3).

The effects of AgNPs, BST and their combination (1:1) on cell viability were tested in HFF1 cells at 24, 48, or 72 h by MTS assay. Data are presented in Figure 14. AgNPs were found to be non-toxic to cells at least up to 15.6 µg/mL concentration after 24 h or 48 h of exposure and up to 31.2 µg/mL concentration after 72 h exposure, whereas AgNPs + BST were non-toxic up to 31.2 µg/mL after 24, 48 or 72 h exposures. Conversely, BST alone had no cytotoxic effect on the cells as cell viability remained 100% at 24, 48 and 72 h of treatment. All experiments were performed in triplicate, and data are presented as mean ± standard deviation of three independent experiments.

## 4. Discussion

Nanofibers have been considered ideal wound healing dressing material due to their multiple pro-wound healing characteristics and by virtue of this they have been commonly used as wound dressings to expedite wound healing [38]. Nanofibers, synthesized using natural or synthetic polymers, demonstrate excellent mechanical strength, allow easy gas exchange at the wound site, and support the sustained and controlled release of loaded bioactive compounds [12]. Moreover, they mimic the skin’s extracellular matrix thereby favoring faster wound healing process [13,14]. Therefore, antibacterial or other pro-wound-healing molecules incorporated into nanofibers or other nanoformulations exercise greater performance compared to that of individual molecules. Among synthetic polymers, PVA demonstrated excellent properties for nanofiber synthesis, as it dissolves easily in water, forms stable solutions, spins smoothly into uniform fibers, is safe, and displays strong mechanical properties. The CS is a naturally derived polymer and is biodegradable and biocompatible, fostering localized and controlled drug delivery.

BST, a common steroid obtained from a variety of plants, has been reported to carry antioxidant, proangiogenic, and anti-inflammatory properties [21,22], as well as antibacterial effects [25,26]. However, these reports are limited and mostly investigated the individual activity of BST (Table 7).

A recent study described the BST- and curcumin-containing oleogel prepared using candelilla wax, to exert antioxidant, anti-inflammatory, antibacterial, and proangiogenic effects in fibroblasts [28]. Likewise, AgNPs exhibit broad-spectrum antimicrobial activity effectively targeting various pathogens, including antibiotic-resistant bacteria (Table 7). Their high surface-area-to-volume ratio enhances their interactions with microbial membranes, disrupting microbial cell membranes, leading to structural alterations and cell death [49,50,51]. Structured bacterial communities called biofilms, which are encased in an extracellular matrix, act as a defense against both the immune system and traditional antibiotics. AgNPs effectively penetrate biofilms and disrupt their structural integrity, preventing bacterial adhesion and colonization. Furthermore, silver ions interfere with bacterial quorum sensing, a communication system that regulates biofilm formation, reducing microbial virulence and persistence [52]. Beyond these mechanisms, AgNPs exert antimicrobial effects through several additional pathways: for example, they form complexes with bacterial DNA and RNA, interfering with replication and transcription, thereby hindering bacterial proliferation [53]. Given the favorable wound healing properties of BST and AgNPs, we constructed, for the first time, a delivery system by incorporating both these agents into PVA–chitosan nanofibers.

The physiochemical properties of the synthesized nanofibers were found to comply with the desired size, morphology, encapsulation efficiency, and time course of release of encapsulated components. The TEM, XRD, and FTIR showed a successful loading of AgNPs and BST into the electrospun PVA/CS/nanofiber matrix. Additionally, PVA/CS/AgNPs/β-S, nanofiber assembly appeared smooth in texture with uniform distribution of spherical AgNPs across the fibers. The AgNPs were spherically dispersed in the fibers with a diameter of 15 nm. The FTIR spectroscopy data confirmed the successful fabrication of PVA/CS nanofiber and incorporation of BST and AgNPs into the fibers without affecting chemical structure. In addition, high encapsulation efficiency and drug release patterns render them to be a potential wound healing system.

In the present study, we employed Gram-positive *Staphylococcus aureus* and Gram-negative *Pseudomonas aeruginosa* bacteria to ascertain the antimicrobial activities of AgNPs and BST either alone or in combination. We found that the MIC values for AgNPs and the AgNPs + BST were 62.5 µg/mL against *S. Aureus* strains ATCC 29213 and ATCC 43300 and 31.2 µg/mL against strain ATCC BAA977. The MIC values for AgNPs and the AgNPs + BST against *P. aeruginosa* strains ATCC 27853 and MDR-7067 were 31.2 and 15.6 µg/mL, respectively, indicating that *P. aeruginosa* strains are more sensitive to AgNPs alone or AgNPs + BST. These data demonstrate and substantiate the antibacterial properties of AgNPs. Conversely, BST failed to exert antibacterial effects against any of the tested strains of *S. aureus* and *P. aeruginosa* across the used concentrations. These data contrast with previous findings where BST was found to exhibit antibacterial effects [25,26,27,28]. This discrepancy possibly stemmed from the differences in the bacterial strains, exposure durations, or BST preparations.

The ZOI test is a reliable method for determining growth inhibitory effects of drugs against multiple bacterial strains. In the present study, the ZOI value of PVA/chitosan nanofibers containing AgNPs + BST was significantly greater than that of AgNPs alone for all the tested bacterial strains except *P. aeruginosa* strain MDR 7067. This implies that the incorporation of AgNPs and BST into nanofibers likely enhanced the bacterial growth inhibitory effect and this observation could be due to the controlled and sustained release of AgNPs facilitated by nanofibers, which corroborates previous findings [54]. Despite BST alone displaying no antibacterial effects against tested bacteria as determined by its MIC values, its contribution to enhanced antibacterial effects cannot be ruled out due to it being a constituent of nanofibers along with AgNPs and nanofibers aiding its controlled and sustained release. In the present study blank PVA/CS nanofibers exhibited antibacterial effects as revealed by ZOI test and these effects were comparable to those of AgNPs alone. These observed antibacterial effects of blank fibers could be attributed to CS as it has been demonstrated previously to execute its antibacterial effect [55,56]. Therefore, the antibacterial activity of AgNPs and BST-containing nanofibers, wherein CS is one of the base constituents of nanofibers along with PVA, could be the result of these three constituents. The augmented antibacterial activity of AgNP + BST-containing nanofibers over that of PVA/CS blank fibers is most likely contributed by AgNPs and BST. This clearly distinguishes the antibacterial activity of PVA/CS-containing blank matrix from that of AgNPs + BST-loaded PVA/CS matrix, particularly due to the fact that PVA lacks an antibacterial effect of its own. This underscores that the observed antibacterial effect of PVA/chitosan/AgNPs/BST assembly is the result of AgNPs + BST as well as chitosan.

To ascertain that AgNPs and BST are not cytotoxic and do not exert any adverse effects on host cells, we exposed HFF1 cells to different concentrations of AgNPs and BST either alone or in combination. These cells treated with AgNPs and AgNPs + BST showed no cytotoxic effect at least up to 15.6 µg/mL and 31.2 µg/mL concentrations respectively, while no cytotoxic effects were seen with BST alone, indicating that compatibility of human cells to AgNPs is dose dependent and is independent at least up to tested dose of BST in the current study. However, it must be noted that the cytocompatibility assessment applied in our study was based on studying the individual or combinatorial effects of AgNPs and BST, rather than examining the effect of the complete nanofiber construct. Additionally, the antibacterial capability of this system was evaluated under an in vitro setting and not under actual wound pathophysiology. In addition, individual contributions of BST towards its antibacterial and other pro-wound healing activities could not be verified.

## 5. Conclusions

In this study, we constructed a novel PVA/chitosan/AgNPs/BST system, which exhibited superior antibacterial effects against both Gram-positive and Gram-negative bacteria, and thus carries significant potential to be used as wound dressing for accelerated wound healing. However, these findings need to be validated through in vivo animal and human studies under actual wound settings.

## Figures and Tables

**Table 1:** Details of different constituents and their concentrations/ratios used in the preparation of nanofibers.

PVA/CS Ratio	Equivalent *v*/*v* Ratio	PVA Volume	CS Volume	PVA Mass	CS Mass	Final PVA Concentration	Final CS Concentration
4:1	80:20	2.4 mL	0.6 mL	240 mg	12 mg	8.0% *w*/*v*	0.4% *w*/*v*
3.5:1.5	70:30	2.1 mL	0.9 mL	210 mg	18 mg	7.0% *w*/*v*	0.6% *w*/*v*
3:2	60:40	1.8 mL	1.2 mL	180 mg	24 mg	6.0% *w*/*v*	0.8% *w*/*v*
2.5:2.5	50:50	1.5 mL	1.5 mL	150 mg	30 mg	5.0% *w*/*v*	1.0% *w*/*v*

PVA—polyvinyl alcohol; CS—chitosan.

**Table 2:** The diameters of the fibers: blank and drug-loaded fibers.

Formulation	Maximum Diameter (nm)	Minimum Diameter (nm)	Average Diameter (nm)
(PVA:CS)	422	287	325.9 ± 35.8
(PVA:CS:BST)	528	372	451.2 ± 37.4
(PVA:CS:AgNPs)	367	222	305.5 ± 35.2
(PVA:CS:BST:AgNPs)	443	329	383 ± 30.8

PVA—polyvinyl alcohol; CS—chitosan; BST—β-sitosterol; AgNPs—silver nanoparticles.

**Table 3:** Comparative FTIR fingerprint.

Sample Peak	Wavenumber (cm^−1^)	Characteristic Peak	Ref.
PVA	3550–3200	Broad O-H stretching	[29]
	3000–2840	C-H stretching (methylene)	
	1750–1730	C=O stretching (residual acetate)	
	1400–1460	C-H_2_ bending	
	1080–1140	C-O/C-O-C stretching	
	920–830	C-C or C-O-H bending	
Chitosan	3450	O-H and N-H stretching	[30]
	2920	C-H stretching	
	1650	C=O amide I	
	1575	N-H amide II	
	1316	C-N mide III	
	1075	C-O stretching	
β-sitosterol	3400	O-H stretching	[31,32]
	2960–2850	C-H stretching	
	1460	CH_2_ bending	
	1313	C-O stretching	
	1055	C-O-C stretching	
Silver nanoparticles	3400	Stretching-OH groups	[33,34]
	2920	C-H stretching	
	1340	C-N stretching	
	1030	C-O stretching vibrations	
	865–825	C-H bending	

**Table 4:** UV–Vis determination of AgNP-loaded fiber content: EE%, and DL.

Sample	EE% (Mean ± SD)	DL (μg/mg) (Mean ± SD)
Fiber 1	87.4 ± 0.1	3.77 ± 0.01
Fiber 2	82.0 ± 0.4	3.55 ± 0.02
Fiber 3	84.4 ± 0.7	3.60 ± 0.03

**Table 5:** EE% and DL of the beta-sitosterol-loaded fiber, expressed as mean ± standard deviation (n = 3).

Sample	EE% (Mean ± SD)	DL (μg/mg) (Mean ± SD)
Fiber 1	63.7 ± 4.5	2.7 ± 0.2
Fiber 2	66 ± 21	2.8 ± 0.9
Fiber 3	70.6 ± 0.1	3 ± 0.01

**Table 6:** The zones of inhibition for five strains of bacteria with AgNP disks, blank fibers, and drug-loaded fibers.

Bacterial Strain	Zone of Inhibition (mm)		
	AgNP’s Disk	Blank Fiber (PVA/Chitosan Only)	AgNPs + BST-Loaded Fiber
*S. aureus* ATCC 29213	10.3 ± 0.5	19 ± 5	34 ± 6 ^a*,b**^
*S. aureus* ATCC BAA 977	10.67 ± 0.5	14 ± 4	23 ± 3 ^a*,b**^
*S. aureus* ATCC 43300	10.3 ± 0.5	19.5 ± 5	32 ± 6 ^a*,b**^
*P. aeruginosa* ATCC 27853	10.3 ± 0.5	16 ± 5.5	26 ± 3 ^a*,b**^
*P. aeruginosa* MDR 7067	13 ± 0	12 ± 4.2	14.5 ± 0.7

^a*^—compared to blank fibers, *p* < 0.05; ^b**^—compared to AgNPs, *p* < 0.01. The data represent mean ± SD of 3 independent experiments (N = 3).

**Table 7:** Details of AgNPs and BST: their synthesis and biological activities.

Antibacterial Agent	Base Components	Synthesis Method	Biological Activity	Ref.
AgNPs	PVA/chitosan hydrogels	Double-crosslinked procedures	Antibacterial	[39]
AgNPs	PVA/chitosan hydrogels	Crosslinking by freeze–thaw	Antibacterial	[40]
Gelatin-capped AgNPs	PVA/PEG/polyaniline hydrogel	Crosslinking by freeze–thaw	Antibacterial	[41]
AgNPs	PVA/chitosan hydrogels	Dried on a film and fabricated	Antibacterial	[42]
AgNPs	Chitosan hydrogel	Physical crosslinking	Antibacterial	[43]
AgNPs	PVA/chitosan	Solvent casting	Antibacterial	[44]
AgNPs	PVA/chitosan sponge	Physical crosslinking	Antibacterial	[45]
AgNPs	PVA, chitosan, PEG, PVP	Physical crosslinking	Antibacterial	[46]
Nano silver	PVA, chitosan	Freeze drying	Antibacterial	[47]
AgNPs	PVA, chitosan, glutaraldehyde	Electrospinning	Antibacterial	[48]
BST + α-pinene	alginate/gelatin hydrogel scaffold	Crosslinking	Antibacterial and antibiofilm	[26]
BST	Ethanolic extract	Formulation	Antifungal and antibacterial	[25]
BST	Direct analysis	Extraction	antibacterial	[27]
BST + Curcumin	Solgel	Formulation	Antioxidant, anti-inflammatory, proangiogenic, and antibacterial	[28]

PVA—polyvinyl alcohol; PEG—polyethylene glycol; PVP—polyvinylpyrrolidone; AgNPs-silver nanoparticles.

## Data Availability

All the data are presented in the manuscript.

## Abbreviations

The following abbreviations are used in this manuscript:

AgNPs	Silver nanoparticles
BST	β-sitosterol
EE	Encapsulation efficiency
FTIR	Fourier-transform infrared spectroscopy
kV	Kilovolts
ƛ	Lambda
MTS	3-(4,5-Dimethylthiazol-2-yl)-5-(3-carboxymethoxyphenyl)-2 (4-sulfophenyl)-2H-tetrazolium
PVA	Polyvinyl alcohol
CS	Chitosan
SEM	Scanning electron microscopy
TEM	Transmission electron microscopy
UV–Vis	Ultraviolet visible spectrophotometer
wt.%	Weight percent
XRD	X-ray diffraction
HFF1	Human foreskin fibroblast
